# An In Vitro Study on the Antimicrobial Properties of Essential Oil Modified Resin Composite against Oral Pathogens

**DOI:** 10.3390/ma13194383

**Published:** 2020-10-01

**Authors:** Barbara Lapinska, Aleksandra Szram, Beata Zarzycka, Janina Grzegorczyk, Louis Hardan, Jerzy Sokolowski, Monika Lukomska-Szymanska

**Affiliations:** 1 Department of General Dentistry, Medical University of Lodz, 92-213 Lodz, Poland; barbara.lapinska@umed.lodz.pl (B.L.); ola.wilzak@gmail.com (A.S.); jerzy.sokolowski@umed.lodz.pl (J.S.); 2 Department of Microbiology and Laboratory Medical Immunology, Medical University of Lodz, 92-213 Lodz, Poland; beata.zarzycka@umed.lodz.pl (B.Z.); janina.grzegorczyk@umed.lodz.pl (J.G.); 3 Department of Restorative Dentistry, Dental School, Saint Joseph University, 11072180 Beirut, Lebanon; louis.hardan@usj.edu.lb

**Keywords:** essential oils, oral pathogens, antibacterial activity, *S. mutans*, *L. acidophilus*, *C. albicans*, antifungal activity

## Abstract

Modifying the composition of dental restorative materials with antimicrobial agents might induce their antibacterial potential against cariogenic bacteria, e.g., *S.* *mutans* and *L.* *acidophilus*, as well as antifungal effect on *C.* *albicans* that are major oral pathogens. Essential oils (EOs) are widely known for antimicrobial activity and are successfully used in dental industry. The study aimed at evaluating antibacterial and antifungal activity of EOs and composite resin material (CR) modified with EO against oral pathogens. Ten EOs (i.e., anise, cinnamon, citronella, clove, geranium, lavender, limette, mint, rosemary thyme) were tested using agar diffusion method. Cinnamon and thyme EOs showed significantly highest antibacterial activity against *S.* *mutans* and *L.* *acidophilus* among all tested EOs. Anise and limette EOs showed no antibacterial activity against *S.* *mutans*. All tested EOs exhibited antifungal activity against *C.* *albicans*, whereas cinnamon EO showed significantly highest and limette EO significantly lowest activity. Next, 1, 2 or 5 µL of cinnamon EO was introduced into 2 g of CR and microbiologically tested. The modified CR showed higher antimicrobial activity in comparison to unmodified one. CR containing 2 µL of EO showed the best antimicrobial properties against *S.* *mutans* and *C.* *albicans*, while CR modified with 1 µL of EO showed the best antimicrobial properties against *L.* *acidophilus*.

## 1. Introduction

Resin composites are the most commonly used dental restorative materials. They are composed of organic matrix and inorganic filler and their properties can be modeled with addition of specific components. The literature provides data on various modifications of dental composites and adhesives performed to enhance their physico-chemical, mechanical and antimicrobial properties [1,2,3,4,5]. Antibacterial activity of monomers, such as 12-methacryloyloxydodecylpyridinium bromide (MDPB), has been widely investigated [5]. Among antibacterial agents introduced into the composition of dental resin materials, most commonly described in the literature are nanoparticles, such as silver, gold, titanium dioxide, zinc oxide or calcium phosphate, as well as fluoride-containing filler and fluoride compounds [6,7,8,9,10,11,12]. Essential oils (EOs) could be promising alternative to contribute to the antimicrobial effect of resin composite materials [13,14].

Essential oils are natural, volatile complex compounds characterized by the odor of their corresponding aromatic plants [15]. There is no systematic chemical nomenclature for chemical compounds found in EOs. However, the scientific names are based on their properties or prominent sources (e.g., limonene, pinene and thymol) [16,17,18]. They exhibit hydrophobic nature and often lower density in comparison to water and are generally lipophilic. Moreover, EOs are soluble in organic solvents, but immiscible with water [19].

EOs are plant products that for decades have been used in traditional healing worldwide. EOs are biosynthesized as secondary metabolites such as bark (cinnamon), buds (clove), flowers (jasmine, rose, violet and lavender), fruits (star anise), herbs, leaves (thyme, eucalyptus and salvia), twigs, wood (sandal), rhizome and roots (ginger), seeds (cardamom) and zest (citrus) [19]. EOs represent a small fraction of plant composition (less than 5% of the vegetal dry matter) and comprise mainly hydrocarbon terpenes (monoterpenes and sesquiterpenes) and terpenoids (isoprenoids). The chemical components of EOs may be produced through either the methylerythritol or the mevalonate or the shikimic acid pathway [19]. Over 100 different components in various ratios (1%–70%) can be found in a single type of EO.

EOs exhibit different biological and pharmacological activities, such as antibacterial, antifungal, antiviral, antimutagenic, antiprotozoal, anti-inflammatory, antidiabetic, antinociceptive, antiphlogistic and antioxidant properties [20,21,22,23,24,25,26,27,28,29,30].

The combination of several EOs may lead to an additive or antagonistic effect against pathogens [31]. The enhanced antibacterial activity of EO mixture in comparison to individual products may result from the synergic effect of EO compounds. This effect relies either on inhibiting common biological pathway in microorganisms, suppressing the protective enzymes, or modifying the functions of the cellular wall [32]. EOs consist of different chemical compounds which may have different antimicrobial modes of action. Therefore, the possibility of antimicrobial resistance is minimized [17].

The mechanism of action of EOs against microorganisms has not been completely understood so far. EOs owe the antimicrobial properties to their volatile components, including terpenoids and phenolic compounds [33]. EO phenolic compounds are known to penetrate through the microbial membrane (formatting pores) leading to the leakage of ions and cytoplasmatic content and finally to cellular breakdown [17,34].

In oral hygiene and dentistry, essential oils are used as components of mouthwashes (i.e., Cool Mint, Listerine Antiseptic, Johnson&Johnson, Skillman, NJ, USA), toothpastes, antiseptic solutions and temporary filing materials (eugenol-based products, i.e., zinc oxide-eugenol cement) [35,36]. Incorporating essential oils into adhesive systems may contribute to the decrease in occurrence of secondary caries due to its antimicrobial activity reported in an *in vitro* microcosm dental biofilm model [14]. The main oral pathogens, *Streptococcus mutans* and *Lactobacillus acidophilus* are crucial in caries development. *S. mutans* plays main role in early demineralization of dental hard tissues, while *L. acidophilus* is pivotal in caries development. Various attempts has been made to enhance antibacterial properties of dental materials, involving the addition of silver-releasing filler [6,7], calcium fluoride [8,12] or amorphous calcium phosphate [9] into the composition of dental resin materials or adhesives. Studies reported that incorporation of essential oil into dental composite structure do not significantly compromise the mechanical properties [13,37], while it could improve its antibacterial activity and thus reduce the risk of secondary caries.

Yeasts, such as *Candida albicans*, are found in oral cavity as structural component of dental plaque biofilm, but more recently it has been recognized as part of cariogenic microbiota [38,39,40,41]. *C. albicans* is capable of producing acids that might demineralize dental hard tissues. According to Nikawa et al. [42], *C. albicans* possesses the ability to dissolve hydroxyapatite to a greater extent (approximately 20-fold) when compared with *S. mutans*. Furthermore, the presence of *streptococci* may promote *C. albicans* colonization of dental hard tissues [43]. Studies suggest symbiotic fungal-bacterial relationship between *S. mutans* and *C. albicans* within the biofilm that prevents from killing or inhibiting each other [44]. However, Maijala et al. [45] claimed that the role of *C. albicans* in cariogenic process is highly overestimated. Incorporating essential oils into dental materials composition seems like a promising alternative that would allow for enhancement of antimicrobial activity of dental restorative materials. In terms of potential anticariogenic effect, it would be favorable to investigate antimicrobial activity of various EOs against major cariogenic pathogens, such as *S. mutans*, *L. acidophilus* and *C. albicans*, in the same study, in homogeneous conditions. That would help to select the most active EOs in order to further incorporate them into dental materials composition to enhance their clinical performance. Thus, the primary aim of this study was to assess which of the different essential oils has the highest antimicrobial activity against oral pathogens (*S. mutans*, *L. acidophilus* and *C. albicans*). Next, the most effective essential oil would be selected to incorporate into resin material and the secondary aim of the study was to evaluate antimicrobial activity against *S. mutans*, *L. acidophilus* and *C. albicans* of the modified resin composite material.

## 2. Materials and Methods

This study used the following ten commercially available essential oils (dr Beta, Pollena Aroma, Nowy Dwór Mazowiecki, Poland): anise, cinnamon, citronella, clove, geranium, lavender, limette, mint, rosemary and thyme. The composition of tested EOs was presented in Table 1, based on data obtained from previous studies analyzing the EOs’ composition by gas chromatography with flame-ionization and mass spectroscopic detection (GC-FID-MS) [46,47,48,49,50,51,52] or data from European Pharmacopoeia [53].

### 2.1. Microbiological Studies of Essential Oils

Microbiological studies were performed on three reference strains: *Streptococcus mutans* ATCC 25175 (Oxoid, Basingstoke, UK), *Lactobacillus acidophilus* ATCC 4356 (Oxoid, Basingstoke, UK) and *Candida albicans* ATTC 10231 (Biocorp, Warsaw, Poland). The colonies were stored in Microbank system (Viabank, Medical Wire&Equipment, Corsham, UK) in the temperature of −30 °C until the experiment was performed. The study protocol was described in detail in previous research paper [8].

Antimicrobial activity of essential oils was tested using agar diffusion test. After 18 h of cultivation, the suspension has been prepared with the turbidity of the 0.5 McFarland standard and inoculated on Mueller–Hinton II Agar medium (Becton Dickinson, Franklin Lakes, NJ, USA) for *S. mutans*, on RPMI 1640 + NaHCO_3_ + L-Glutamine + phenol red medium (Biocorp, Warsaw, Poland) for *C. albicans* and media consisting of 90% IST (Oxoid, Basingstoke, UK) agar and 10% MRS (Oxoid, Basingstoke, UK) agar adjusted to pH 6.7 for *L. acidophilus*.

An automatic micropipette (Proline^®^ Plus 2–20 μL, Sartorius Biohit Liquid Handling Oy, Helsinki, Finland) was used to apply 6 μL of tested essential oil on filter paper discs (Oxoid, Basingstoke, UK). Chlorhexidine digluconate (CHX) aqueous solution (0.2%) served as a positive control. Filter paper discs (6 mm in diameter) were incubated for 20 min in room temperature in order to ensure the homogenous absorption of tested essential oil. Blank discs were used as negative control.

Next, sterile filter paper discs with tested oils, CHX and blank ones were placed directly on the inoculated agar surface. Special care was taken to ensure uniform contact of the paper disc with the media surface. The cultures were incubated for 18 h at temperature of 35 °C: for *S. mutans* in CO_2_ enriched conditions—GENbox CO_2_ (bioMerieux S.A., Marcy l’Etoile, France), for *L. acidophilus* in anaerobic conditions; GENbox anaer (bioMerieux S.A., Marcy l’Etoile, France), for *C. albicans—*in aerobic conditions. After the removal of paper discs, the inhibition growth zones were measured (without subtracting disc diameter). For each tested EO and CHX, twelve filter paper discs were used to measure inhibition growth zone of every tested strain.

### 2.2. Microbiological Studies of Composite Resin Material Modified with Essential Oil

The chosen essential oil was introduced into flowable bulk-fill composite resin (CR) material (SDR flow, Dentsply Sirona, Konstanz, Germany) and mixed mechanically until obtaining uniform and homogenous consistency. The essential oils and dimetacrylate resins possess hydrophobic features hence they can be easily mixed to obtain homogeneous material. The material was modified with the essential oil that exhibited the highest antimicrobial activity. The concentrations of the essential oil were chosen as follows: Group 1: 1 µL of EO in 2 g of CR; Group 2: 2 µ of EO in 2 g of CR; Group 3: 5 µL of EO in 2 g CR.

Disc-shaped (3 mm of height and 6 mm in diameter) samples of composite resin material modified with essential oil were performed. Each sample was light-cured with halogen lamp (Megalux Soft-start, Mega-PHYSIC Gmbh & Co. KG, Rastatt, Germany) according to the manufacturer’s instruction (i.e., 20 s). To evaluate antimicrobial activity against *S. mutans*, *L. acidophilus* and *C. albicans* of essential oil modified composite resin (EO-CR) eluate method was used.

The samples were placed in 2.5 mL of 0.95% NaCl solution and incubated for 24 h in temperature of 35 °C. Next, after removing tested samples from the eluate, serial dilutions of the tested microbial strains were prepared (10^−1^, 10^−2^, 10^−3^, 10^−4^, 10^−5^ and 10^−6^) by the introduction of 200 µL of the strains into 1.8 mL of eluate. Strains were incubated for 18 h.

The control group was a sample of composite resin material, not modified with essential oil, that was incubated in the same conditions as the study groups samples.

After the incubation, to evaluate bacterial susceptibility, 100 µL of the control and 100 µL of bacteria dilution (of each dilution) in eluate were cultivated as follows: *S. mutans* on MH agar medium (Becton-Dickinson, Franklin Lakes, NJ, USA) in CO_2_-enriched conditions—GENbox CO_2_ (bioMerieux S.A., Marcy l’Etoile, France); *L. acidophilus* in anaerobic conditions on GENbox anaer medium (bioMerieux S.A., Marcy l’Etoile, France), and *C. albicans* in aerobic conditions on RPMI 1640 medium (Thermo Fisher Scientific, Waltham, MA, USA). The strains were incubated for 24 h in temperature of 35 °C.

Upon the cultivation period, bacterial colonies in the studied samples and the control group were counted. The experiment was repeated twelve times for each EO-CR group and for the control group.

### 2.3. Statistical Analysis

The descriptive analysis of numerical variables encompasses the calculation of the mean (M) along with standard deviations (SD) values. The statistical analysis of the significance consisted of the following: Shapiro–Wilk W test for normality; Levene’s tests for equality of variances; One-way analysis of variance; Kruskal–Wallis equality-of-populations rank test; Post-hoc multiple comparison tests; Zero-inflated Poisson regression with robust standard errors. A level of *p* < 0.05 was deemed statistically significant. The statistical analyses of were carried out using Stata^®^/Special Edition, release 14.2 (StataCorp LP, College Station, TX, USA). The post-hoc statistical power was calculated using post-hoc power analysis calculator (https://clincalc.com/stats/Power.aspx) and a statistical power of 98.56% was found.

## 3. Results

### 3.1. Antimicrobial Activity of Essential Oils (Inhibition Growth Zone)

**Figure 1:**
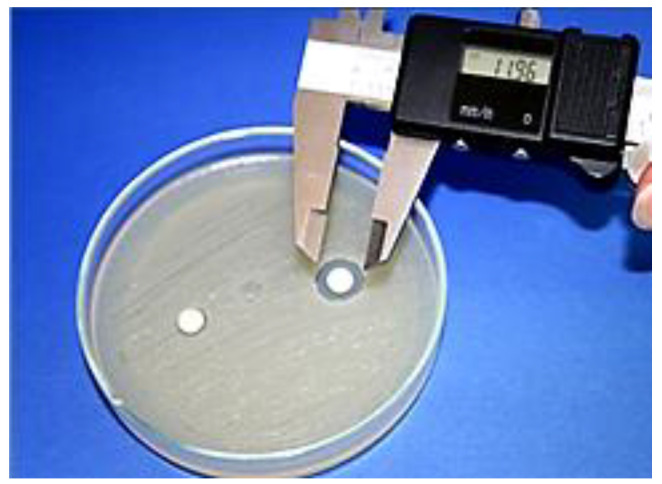
Representative figure of measurement of growth inhibition zone.

**Figure 2:**
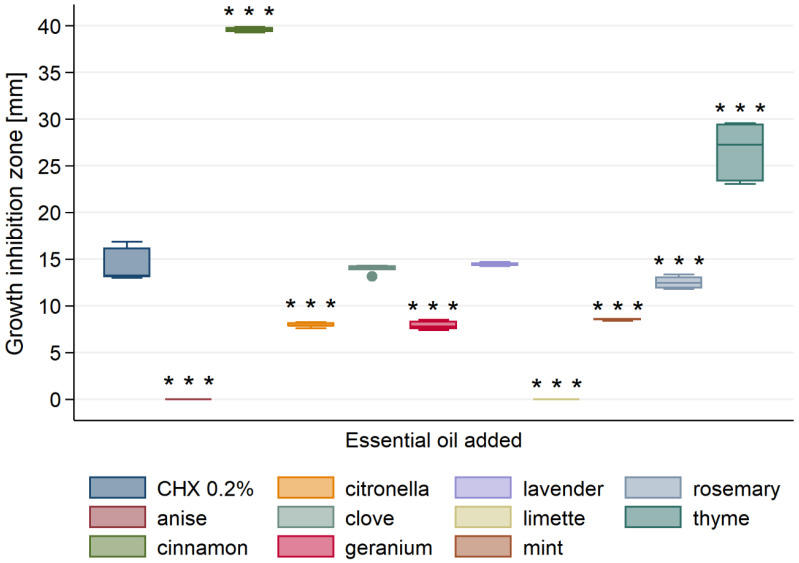
Antibacterial activity of tested essential oils against *S. mutans*. *** *p* < 0.001 versus positive control (0.2% CHX).

**Figure 3:**
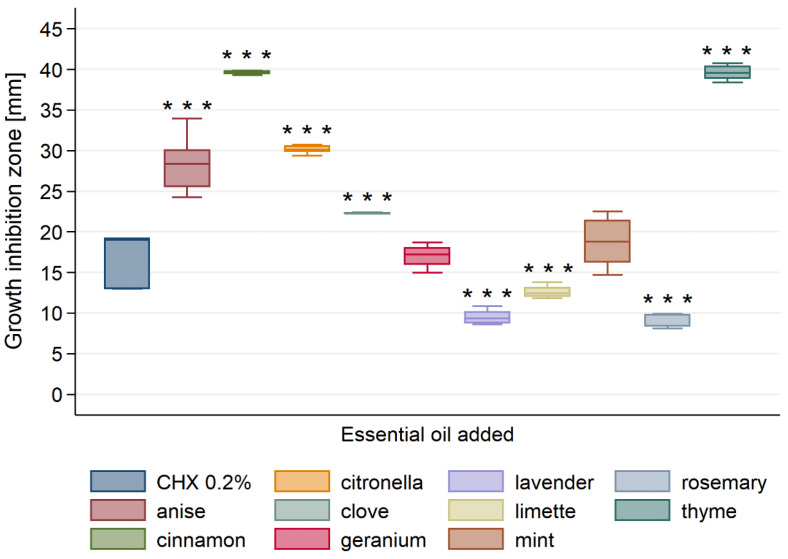
Antibacterial activity of tested essential oils against *L. acidophilus*. *** *p* < 0.001 versus positive control (0.2% CHX).

**Figure 4:**
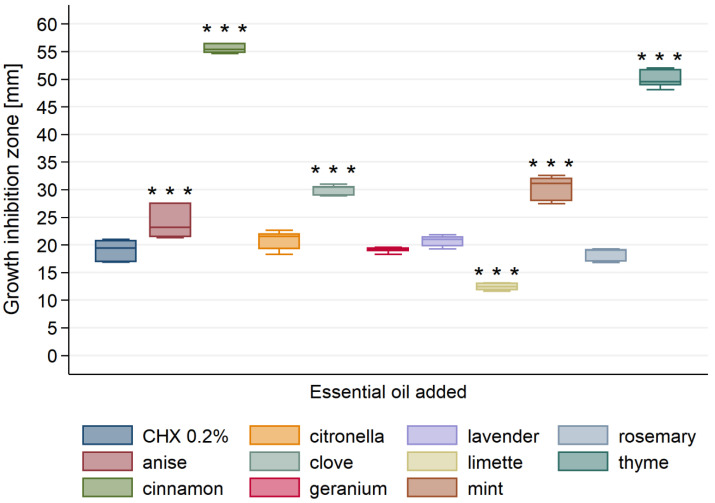
Antifungal activity of tested essential oils against *C. albicans*. *** *p* < 0.001 versus positive control (0.2% CHX).

Figure 1 shows a measurement of representative inhibition growth zone of tested EO. The inhibition growth zones of tested microbes measured for each essential oil were presented in Figure 2, Figure 3 and Figure 4. All tested essential oils, with exception to anise and limette EOs, were found to possess antibacterial activity against *S. mutans* (Figure 2). The diameter of the inhibition zone of *S. mutans* ranged from 0 mm for anise and limette essential oils up to 40 mm for cinnamon essential oil.

The cinnamon oil showed significantly highest antibacterial activity among all ten tested essential oils. Next, it was the thyme EO that exhibited significantly higher activity than anise, citronella, clove, geranium, lavender, limette, mint and rosemary EOs, but significantly lower than the cinnamon EO. Clove and lavender EOs exhibited antibacterial activity comparable to the one of 0.2% CHX. Citronella, geranium and mint showed significantly lower activity than other EOs, with exception to anise and limette EOs (Table A1). The latter showed the lowest antibacterial activity against *S. mutans* among all EOs tested.

All tested essential oils were found to possess antibacterial activity against *L. acidophilus* (Figure 3).

The diameter of the inhibition zone of *L. acidophilus* bacteria ranged from 8 to 40 mm. Again, significantly highest antibacterial activity among all tested essential oils showed cinnamon and thyme EOs, followed by anise and citronella EOs. Geranium, mint EOs and CHX showed significantly higher activity than lavender, limette and rosemary EOs, but significantly lower—than anise, cinnamon, citronella, clove and thyme EOs (Table A2). Lavender and rosemary EOs exhibited the significantly lowest antibacterial activity.

All tested essential oils were found to possess antifungal activity (Figure 4). The diameter of the inhibition zone of *C. albicans* ranged from 12 to 56 mm.

The significantly highest antifungal activity among all tested essential oils showed cinnamon EO, followed by thyme EO. Clove and mint EOs showed significantly higher activity than other EOs (with exception to cinnamon and thyme EOs). Citronella, geranium and lavender EOs exhibited significantly lower activity than anise, cinnamon, clove, mint and thyme EOs (Table A3). CHX possessed similar antifungal activity as citronella and lavender EOs. Limette exhibited the significantly lowest antifungal activity among all tested EOs.

### 3.2. Antimicrobial Activity of Composite Resin Modified with Essential Oil

The highest antimicrobial activity against all tested pathogens showed cinnamon EO, hence it was introduced into composite resin material. Next, the modified material was tested for antimicrobial activity against oral pathogens, i.e., *S. mutans*, *L. acidophilus* and *C. albicans*.

**Figure 5:**
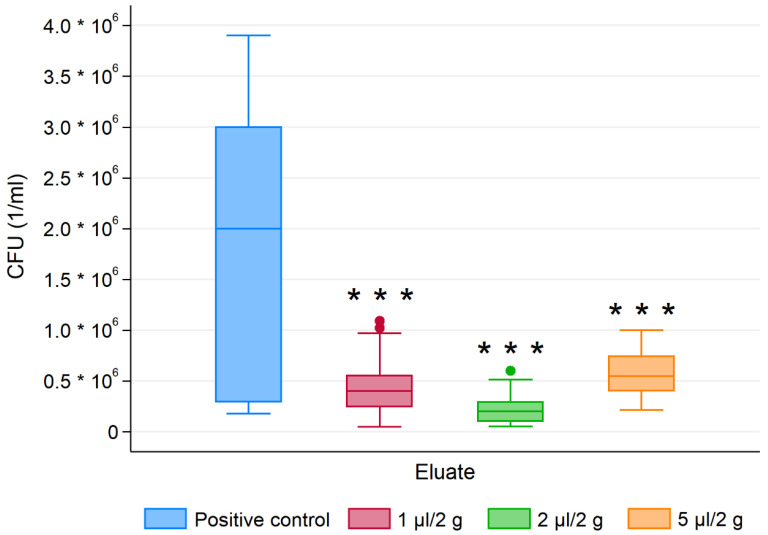
Colony forming units (CFU) of *S. mutans* for essential oil modified composite resins and the control group. *** *p* < 0.001 versus control.

**Figure 6:**
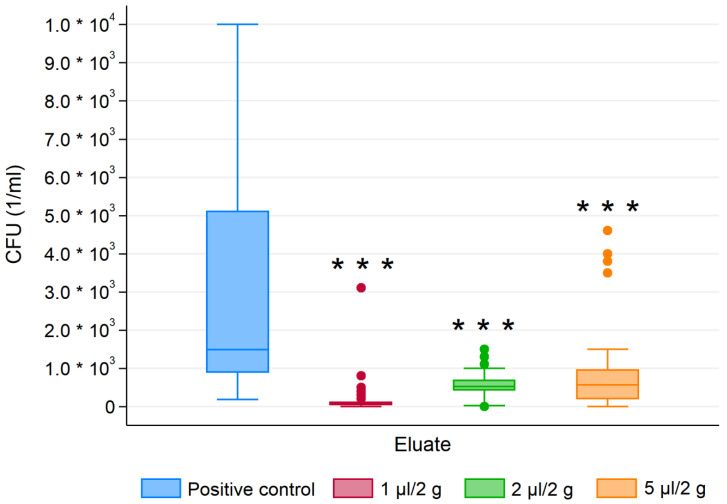
Colony forming units (CFU) of *L. acidophilus* for essential oil modified composite resins and the control group. *** *p* < 0.001 versus control.

**Figure 7:**
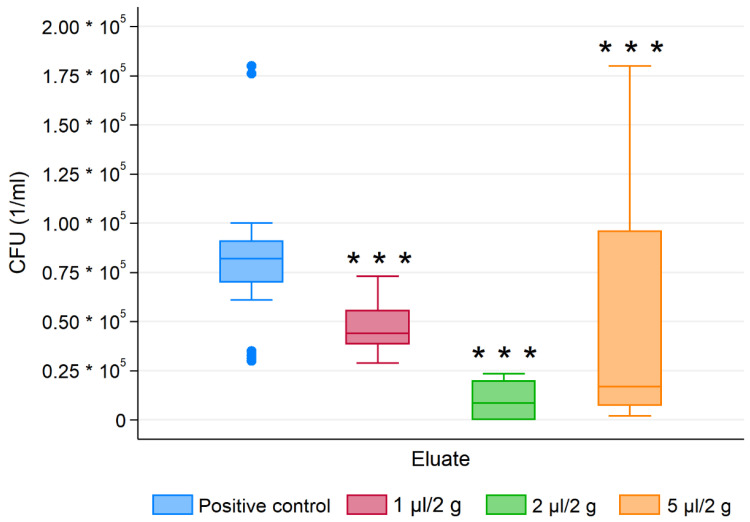
Colony forming units (CFU) of *C. albicans* for essential oil modified composite resins and the control group. *** *p* < 0.001 versus control.

For all tested microbes, the essential oil modified composite resins showed statistically significant different CFU than for the control group regardless of the EO concentration (Figure 5, Figure 6 and Figure 7). Antimicrobial activity of EO-CRs was significantly higher than that of unmodified CR. Furthermore, Fisher’s post-hoc test revealed, that for each tested oral pathogen, the differences in CFU between the study groups were statistically significant.

As for *S. mutans*, the significantly highest antibacterial activity showed 2 µL/2 g EO-CR, followed by 1 µL/2 g EO-CR and 5 µL/2 g EO-CR (*p* < 0.001). Whereas, for *L. acidophilus* the least CFU were noted for 1 µL/2 g EO-CR, followed by 2 µL/2 g EO-CR and 5 µL/2 g EO-CR (*p* < 0.001).

As far as antifungal activity against *C albicans* was concerned, the highest activity showed 2 µL/2 g EO-CR and the lowest 5 µL/2 g EO-CR (*p* < 0.001).

## 4. Discussion

Essential oils have been used in many walks of life, including dentistry. Researchers constantly search for new possibilities of application of effective formulas into dental products. EOs seem to be the promising ingredients of future oral care products and dental materials used both by patients and dentists. The present study investigated antibacterial activity of ten essential oils against three cariogenic pathogens: *S. mutans*, *L. acidophilus* and *C. albicans*. Such great variety of essential oils tested in one study seemed advantageous as the experiment was performed in the same standardized conditions and allowed for reliable assessment and comparison of EOs’ antimicrobial properties. As far as oral pathogens are concerned, most of the previous studies described only a few essential oils [13,14,37,54,55] against one or two most cariogenic bacteria in one setting [56,57].

In the present study, among ten tested essential oils, the most prominent antimicrobial activity exhibited two EOs: cinnamon and thyme. The other EO that showed both significant antibacterial and antifungal effect was clove oil. These results confirmed other findings that EOs possessed potent antibacterial activity and antifungal properties against oral pathogens, including cariogenic bacteria [54,56,57,58]. The study used *S. mutans* and *L. acidophilus*, due to their undisputable involvement in the carious process. The former one is responsible for the initiation of the process and the latter for its development [59,60,61]. Given their proven cariogenic activity, *S. mutans* as well as *Lactobacillus spp*., have been used in the present study. In addition, *C. albicans* is considered to play a supportive role in cariogenic process [42]. Similarly to other studies [62], current study used 0.2% chlorhexidine digluconate aqueous solution as a positive control due to its proved antimicrobial and antifungal activity [63,64].

The composition of EOs determines their antibacterial potential. The highest activity of EOs is provided by thymol, eugenol and carvacrol content, followed by alcohol-containing EOs, with alcohols such as citronellol, geraniol, linalool, menthol, terpinen-4-ol and α-terpineol. Another bioactive group comprise of EOs that contain either ketones, e.g., camphor, carvone, menthone, or thujene or aldehyde groups, i.e., cinnamaldehyde, as well as those with other functional groups, such as anethole and cineole.

Cinnamon essential oil has high percentage of aldehydes (cinnamon aldehydes), that possess antifungal, anti-inflammatory and disinfectant qualities [65]. The effectiveness of cinnamon EO and cinnamon aldehyde against *S. mutans*, *S. mitis*, *S. salivarius*, *A. actinomycetemcomitans*, *P. gingivalis* and *Fusobacterium nucleatum* was reported by Zainal-Abidin et al. [66]. Other studies also confirmed antibacterial activity of cinnamon [47,67] and clove [47,68,69] essential oils against *S. mutans.* High antibacterial activity of clove EOs depends on its aromatic compound content: eugenol (85.3%). Eugenol was reported to have antiseptic, antimicrobial, anesthetic, analgesic, antioxidant, anti-inflammatory and cardiovascular activities [70]. In the present study, cinnamon EO showed significantly higher antimicrobial activity than clove EO, which is consistent with other study [69]. Clove and cinnamon were found to inhibit fungal growth at a concentration of 6% [57]. The results of the present study are consistent with other findings [71] reporting cinnamon oil to have the most potential antibacterial properties. Another study [72] proved cinnamon essential oil to possess the highest antibacterial activity against *S. mutans* among other nine oils (eucalyptol, lime, clove, mint, vinegar, cedar and citrus grass). In addition, Arora and Kaur [73] observed the antimicrobial activity of clove EO against *C. albicans*. It was confirmed by the present study, in which clove EOs exhibited significantly higher activity against *C. albicans* and *L. acidophilus* than CHX, whereas no significant difference between in activity of clove EO and CHX against *S. mutans* was found.

Thyme EO was reported to show antimicrobial activity against oral pathogens due to high content of thymol (38.1%) and *p*-cymene (29.1%) [47,74]. Phenolic compound—thymol, the main component of thyme EO—is reported to disintegrate the outer membrane of Gram-negative bacteria and make bacterial cytoplasmic membrane more permeable to ATP [75]. Another constituent of thyme EO—carvacrol—is proved to exhibit antibacterial potential against *S. mutans* and *C. albicans* [54,62]. Carvacrol emulsion might be also a promising alternative to NaOCl in irrigation of dental root canal system and eradication of intracanal bacteria: *E. feacalis* [76]. Studies proved also a potent antimicrobial activity of thymol against *S. mutans* and *C. albicans* [54,57], as well as against *Porphyromonas gingivalis* and *A. actinomycetemcomitans*, which play a role in development of periodontal disease [77]. That was confirmed by the present study. Thyme EO exhibited the significantly highest antimicrobial activity against *C. albicans* and *S. mutans*, whereas the antibacterial activity against *L. acidophilus* was significantly higher than of other EOs, but lower than that of cinnamon EO. Another study stated that clove, thyme, cinnamon and peppermint EOs are potent antimicrobial phenols [17].

Other EOs tested in the study, such as citronella, geranium, lavender, limette, mint, rosemary presented medium antibacterial activity that is associated with the content of citronellol and geraniol, linalool and linalyl acetate, 1.8-cineole, camphor and α-pinene [74]. As for anise EO, it showed no antibacterial activity against *S. mutans*, whereas its activity against *L. acidophilus* and antifungal activity were high. Antifungal potential of this EO can be attributed to high content of *trans*-anethole, which can interact with fungal plasma [78].

The positive correlation between antibacterial activity of EOs and high content of certain components was reported only for few EOs (e.g., mint, thyme and oregano). For others (e.g., limette and lavender), it is most likely that their antibacterial potential is the result of synergistic effect of the components, since some of those major components exhibit higher antibacterial effect than the EO itself [79].

The present study proved that cinnamon and thyme followed by clove EOs exhibited significantly higher or equal antimicrobial properties against oral pathogens than CHX. These findings would be the introduction to further investigations aiming at the incorporation of these oils into oral care products, i.e., tooth pastes, mouth rinses. The antimicrobial potential of these EOs might be also used to enhance the antibacterial properties of dental materials such as dental resin materials, temporary dressings, disinfectants or root canal filling materials. Furthermore, extracting the most active components from EOs and introducing them into dental products (e.g., restorative materials) composition might be promising line of research. The abundance literature reported that there is great need for development of dental materials with antibacterial properties [4,8,10,12,55,80]. The results of previous studies on antibacterial properties of dental materials seemed promising and suggested that introducing antimicrobial agents into the composition of dental materials might improve their antibacterial potential without deteriorating the physico-mechanical performance [8,12,37].

Given the highest antimicrobial activity obtained in the present study, cinnamon essential oil was used to incorporate, in three different concentrations, into composite resin material composition. Based on preliminary experiments performed, the tested concentration of the EO in composite resin was established as 1, 2 or 5 µL of EO in 2 g of CR. The best antimicrobial properties against *S. mutans* and *C. albicans* were achieved for composite resin containing this essential oil in concentration 2 µL/2 g, whereas against *L. acidophilus* in concentration 1 µL/2 g. Ideally, the composite resin material would present antimicrobial effect and possess very good mechanical properties. The addition of antibacterial or antifungal agents should not change the mechanical performance of the resin material. The current experiment showed that the addition of 2 µL of cinnamon essential oil into 2 g of composite material allows for limiting microbial growth of tested oral pathogens in comparison to unmodified material. This composition might be optimal in terms of antimicrobial properties due to mild influence on polymerization process and enabling release of active compounds into environment.

Still, the present study has some limitations. First of all, the study used single-species model with isolated strains of specific oral pathogens tested in in vitro conditions, without saliva involvement, whereas oral cavity is complex environment holding variety of pathogens interacting in formation of oral biofilm on hard dental tissues. Therefore, these findings must be confirmed in further microbiological studies.

Next, mechanical properties of composite resins modified with essential oils should be tested if considering such materials for clinical application. One study [13] tested mechanical properties of composite resin material modified with cinnamon EO, such as hardness, tensile and flexural strength. The results of the study provided inconsistent data on the proper concentration of the EO in the CR to obtain desirable mechanical performance of the EO-CR material. However, the addition of cinnamon EO to composite material did not adversely affect all the mechanical properties. CR material showed significantly higher flexural strength when modified with 1 µL of cinnamon EO (in 2 g of the material) than non-modified CR. In contrast, non-modified CR showed significantly higher hardness (HV1) and tensile strength values in comparison to modified CR. As far as tensile strength of EO-CR material was concerned, the addition of 2 µL of cinnamon EO (in 2 g of the material) allowed for obtaining significantly highest results. On the contrary, the addition of high amount of EO (5 µL/2 g) significantly deteriorated all tested mechanical properties. Still, such EO-modified bulk-fill material could be clinically used in pediatric dentistry as a final filling in primary teeth or in permanent teeth as a temporary filling, as a liner or in two-step bulk restorative technique in deep cavities. Furthermore, class V cavities, with minimum occlusal loading could be restored with such composite material. Still, long-term performance of such restorations and their aesthetic features must be evaluated.

Moreover, long term study should be performed to evaluate possible allergic reaction to essential oil modified composite resin material as well as the cytotoxic effect of EOs released from EO-CRs. Study showed that EOs present cytotoxic effects on living cells and the severity depends on their type and concentration [16]. Hence, further studies should be conducted to evaluate the potential cytotoxicity and long-term antimicrobial effect of essential oils incorporated into the dental restorative materials.

Since the present study tested only one restorative material, the results cannot be translated to other composites resin materials due to some variation in their composition.

## 5. Conclusions

The study showed that all ten tested essential oils possess antibacterial activity against *L. acidophilus* and antifungal activity against *C. albicans*. Only two essential oils, anise and limette were ineffective towards *S. mutans*. Among tested essential oils, the cinnamon and thyme showed overall the highest antibacterial and antifungal activity against oral pathogens used in the study. Composite resin modified with cinnamon essential oil showed antimicrobial effect regardless of the EO concentration. Considering these preliminary results, essential oils seem promising alternative to other antibacterial agents incorporated into resin composite and further studies should be conducted to further evaluate the antimicrobial effect of dental composites modified with essential oils, as well as their mechanical properties.

## Figures and Tables

**Table 1:** Characteristics of essential oils used in the study.

Essential Oil (Name of EO in INCI)	Composition
Star anise (*Illicium Verum Oil*)	*trans*-anethole (86.0%–93.0%), linalool (0.2%–2.5%), estragole (0.5%–6.0%), α-terpineol (<0.3%), *cis*-anethole (0.1%–0.5%), anisaldehyde (0.1%–0.5%), foeniculin (0.1%–3.0%) [53]
Cinnamon (*Cinnamomum Zeylanicum Bark Oil*)	cinnamaldehyde (76.8%), methoxycinnamaldehyde (11.7%), cinnamyl acetate (3.2%), cumarin (1.5%), benzaldehyde (1.1%) [48,49]
Citronella (*Cymbopogon Winterianus Oil*)	citronellal (36.2%), geraniol (22.4%), citronellol (14.1%), limonene (3.5%), elemol (3.3%), citronellyl acetate (3.2%) [51]
Clove (*Eugenia Caryophyllus Oil*)	eugenol (85.3%), β-caryophyllene (10.6%), α-humulene (2.0%) [47,49]
Geranium (*Pelargonium Graveolens Oil*)	citronellol (26.7%), geraniol (13.4%), nerol (8.7%), citronellyl formate (7.1%), isomenthone (6.3%), linalool (5.2%), 10-*epi*-γ-eudesmol (4.4%), geranyl formate (2.5%), menthone (1.6%), β-caryophyllene (1.5%), geranyl butyrate, *cis*-rose oxide (1.4%), geranial (1.1%), β-baurobonene (1.1%) [47,48,49,52]
Lavender (*Lavandula Angustifolia Oil*)	linalool (34.1%), linalyl acetate (33.3%), lavandulil acetate (3.2%), β-ocymene (3.2%), β-caryophyllene (2.7%), cineole (2.5%), terpinen-4-ol (2.5%), myrecene (2.4%), α-terpineol (1.8%) [48,49]
Limette (*Citrus aurantifolia oil*)	linalyl acetate (48.06%), linalool (26.88%), α-terpineol (5.74%), geranyl acetate (3.92%), geraniol (3.05%), geranial (2.44%) [50]
Mint (*Mentha Piperita Oil*)	menthol (30.0%–55.0%), menthone (14.0%–32.0%), cineole (3.5%–14.0%), menthyl acetate (2.8%–10.0%), isomenthone (1.5%–10.0%), menthofuran (1.0%–9.0%), limonene (1.0%–5.0%), isopulegol (<0.2%), pulegone (<4.0%), carvone (<1.0%) [53]
Rosemary (*Rosmarinus Officinalis Oil*)	1.8-cineole (46.4%), camphor (11.4%), α-pinene (11.0%), β-pinene (9.2%), camphene (5.2%), β-caryophyllene (3.5%), borneol (3.1%), αa-terpineol (1.8%), *p*-cymene (1.3%), myrecene (1.2%) [47,49]
Thyme (*Thymus Vulgaris Oil*)	thymol (38.1%), *p*-cymene (29.1%), γ-terpinene (5.2%), linalool (3.7%), β-Caryophyllene (3.1%), carvacrol (2.3%) [46,47,49]

Legend: INCI = International Nomenclature of Cosmetic Ingredients.

## Appendix A

**Table A1:** Levels of statistical significance (*p*) in Fisher’s post-hoc test of EOs activity against *S. mutans.*

Essential Oil	Anise	Citronella	Cinnamon	Clove	Geranium	Lavender	Limette	Mint	Rosemary	Thyme
**Anise**		NS	NS	NS	NS	NS	NS	NS	NS	NS
**Citronella**								0.688		
**Cinnamon**		**<0** **.** **001**		**<0.001**		**<0.001**		**<0.001**	**<0.001**	
**Clove**		**<0.001**				0.157		**<0.001**		
**Geranium**		0.905	**<0.001**	**<0.001**		**<0.001**	NS	0.604	**<0.001**	**<0.001**
**Lavender**		**<0.001**						**<0.001**		
**Limette**		NS	NS	NS		NS		NS	NS	
**Mint**										
**Rosemary**		**<0.001**		0.181		0.940		**<0.001**		
**Thyme**		**<0.001**	**<0.001**	**<0.001**		<0.001	NS	**<0.001**	**<0.001**	

NS = not significant.

**Table A2:** Levels of statistical significance (*p*) in Fisher’s post-hoc test of EOs activity against *L. acidophilus.*

Essential oil	Anise	Cinnamon	Citronella	Clove	Geranium	Lavender	Limette	Mint	Rosemary	Thyme
**Anise**		**<0.001**	0.544	**<0.001**	**< 0.001**	**<0.001**	**<0.001**	**<0.001**	**<0.001**	**<0.001**
**Cinnamon**			**<0.001**	**<0.001**		**<0.001**		**<0.001**	**<0.001**	
**Citronella**								**<0.001**		
**Clove**			**<0.001**			**<0.001**		**<0.001**		
**Geranium**		**<0.001**	**<0.001**	**<0.001**		**<0.001**	**<0.001**	0.209	**<0.001**	**<0.001**
**Lavender**			**<0.001**					**<0.001**		
**Limette**		**<0.001**	**<0.001**	**<0.001**		**<0.001**		**<0.001**	**<0.001**	
**Mint**										
**Rosemary**			**<0.001**	**<0.001**		0.557		**<0.001**		
**Thyme**		0.734	**<0.001**	**<0.001**		**<0.001**	**<0.001**	**<0.001**	**<0.001**	

**Table A3:** Levels of statistical significance (*p*) in Fisher’s post post-hoc test of EOs activity against *C. albicans*

Essential oil	Anise	Cinnamon	Citronella	Clove	Geranium	Lavender	Limette	Mint	Rosemary	Thyme
**Anise**		**<0.001**	**<0.001**	**<0.001**	**<0.001**	**<0.001**	**<0.001**	**<0.001**	**<0.001**	**<0.001**
**Cinnamon**			**<0.001**	**<0.001**		**<0.001**		**<0.001**	**<0.001**	
**Citronella**								**<0.001**		
**Clove**			**<0.001**			**<0.001**		0.486		
**Geranium**		**<0.001**	0.030	**<0.001**		**0.001**	**<0.001**	**<0.001**	0.648	**<0.001**
**Lavender**			0.252					**<0.001**		
**Limette**		**<0.001**	**<0.001**	**<0.001**		**<0.001**		**<0.001**	**<0.001**	
**Mint**										
**Rosemary**			0.009	**<0.001**		**<0.001**		**<0.001**		
**Thyme**		**<0.001**	**<0.001**	**<0.001**		**<0.001**	**<0.001**	**<0.001**	**<0.001**	
